# Examining epidemiological models and economic analyses of typhoid conjugate vaccine: A scoping review

**DOI:** 10.1371/journal.pgph.0005162

**Published:** 2026-03-30

**Authors:** Prashant Mandaliya, Stacey Orangi, Jacob Kazungu, Dennis Waithaka, Angela Kairu, Amos Bationo, Felix Masiye, Obinna Onwujekwe, Edwine Barasa

**Affiliations:** 1 Health Economics Research Unit, KEMRI Wellcome Trust Research Programme, Nairobi, Kenya; 2 Department of Economics, School of Humanities and Social Sciences, University of Zambia, Zambia; 3 Departments of Health Administration & Management and Pharmacology & Therapeutics, College of Medicine, University of Nigeria, Nsukka, Nigeria; 4 Centre for Global Health and Tropical Medicine, Nuffield Department of Medicine, University of Oxford, Oxford, United Kingdom; PLOS: Public Library of Science, UNITED STATES OF AMERICA

## Abstract

Typhoid remains a public health concern across many low- and middle-income countries. This scoping review summarized and mapped evidence from epidemiological models and economic analyses of typhoid conjugate vaccine (TCV). We included studies on the cost of illness of typhoid, the cost of vaccination, cost-effectiveness, and public health impact of TCV. A search was conducted across five databases and followed the Arksey and O’Malley’s methodological framework. We extracted data on the study design, population, outcomes, model parameters, and cost-effectiveness. All reported costs were converted to 2024 United States Dollars (USD) to allow for comparisons across studies. Findings were summarized by key outcomes. The 26 studies included in the review covered Sub-Saharan Africa, South and East Asia, the Pacific, Latin America, the Middle East, and Europe. A total of ten studies reported the cost of treating a typhoid patient, with costs varying based on the hospital setting, geographic location and severity of the disease. Five studies examined the cost of TCV vaccination and delivery reported higher costs for campaigns than routine immunization, with economic costs consistently higher than financial costs. The two public health impact studies predicted case reductions from 2% to 94% and mortality reductions up to 36% or 100%, depending on the vaccine strategy employed. The nine economic evaluations included in the review reported lower incremental cost effectiveness ratios in high-incidence urban settings, with some strategies termed as cost-saving. Cost-effectiveness depends on incidence, perspective, and vaccine strategy, emphasizing the need for context-specific evaluations. Literature shows that the value of TCV is influenced by the disease burden, vaccination costs, and health outcomes, which are context specific. Future research should employ more comprehensive models to better capture TCV’s economic and public health value and leverage on local data to give more contextual evidence for policymakers.

## Introduction

Typhoid fever, caused by *Salmonella enterica serovar Typhi* (*S. Typhi*) remains a public health challenge in low- and middle-income countries (LMICs). It is transmitted primarily through ingestion of contaminated food or water [1]. Some individuals become chronic carriers and continue to shed the bacteria for years, resulting in continuous transmission [1]. Typhoid is endemic in several regions, including Africa, Asia, Eastern Mediterranean, Latin America, Oceania, and the Western Pacific [2]. Countries with incidence rates exceeding 100 cases per 100,000 persons per year are considered as highly endemic [2]. According to the Global Burden of Disease Study 2021, South Asia (379.6 per 100,000 person-years (95% UI: 295.9-484.0)) and Sub-Saharan Africa (94.8 per 100,000 person-years (95% UI: 73.8-121.6)) had the highest incidence [3]. Recent systematic reviews reported the highest typhoid incidence in children, globally [4,5], particularly those aged 5–9 years in Asia and 10–14 years in Africa [6]. The disease caused an estimated 108,000 deaths globally in 2021 [3].

Early initiation of treatment with appropriate antibiotics can reduce case-fatality rate to 1–4%, compared to 20% in untreated cases [7]. The rise of multidrug-resistant *S. Typhi* strains, including resistance to fluoroquinolones and azithromycin, threatens disease control [8]. Limited treatment access in LMICs and increasing antimicrobial resistance underscores the importance of preventive interventions such as vaccination, surveillance, behavioral changes, and improved sanitation [9]. In 2017, the World Health Organization’s (WHO) Strategic Advisory Group of Experts (SAGE) on Immunization recommended introducing typhoid conjugate vaccine (TCV) to routine immunization programs in endemic countries [10]. WHO has approved three typhoid vaccines, TCV, unconjugated Vi polysaccharide (ViPS), and live attenuated Ty21a [11]. While the ViPS and Ty21a vaccines have been available since the early 2000s, their uptake has been limited [11,12]. TCV offers multiple advantages, including greater vaccine efficacy (>80%), longer duration of protection, a single-dose schedule, and suitability for children from six months of age [13–16]. These benefits make TCV a strong candidate for routine immunization in LMICs [11,13–20].

Evidence on the economic impact of TCV is still limited, yet economic evaluations can provide valuable insights for public health decision-making. These include cost-of-illness (COI) studies, which quantify the total economic burden of a disease [21]. In addition, vaccine delivery cost studies estimate the cost of delivering the vaccine to the target population (excluding procurement costs of the vaccine and supplies), and cost of vaccination studies estimate the total cost of procuring and delivering the vaccine, accounting for both financial and economic resources used for vaccination [22,23]. Modeling studies also play a vital role by simulating potential long-term outcomes of different vaccination strategies [24]. Cost-effectiveness analysis (CEA) further compares the costs and health outcomes to determine the economic value of interventions and guide resource allocation [24]. Together, these components provide a comprehensive understanding of the economic and societal value that vaccines offer, supporting immunization programs to achieve the most significant public health impact.

A review by Luthra et al. in 2019 offered insights into the cost of treating typhoid, but only identified two published studies on TCV, both of which were cost-effectiveness analyses [24]. However, since the WHO SAGE recommendation and prequalification of TCV in 2018, along with the Global Alliance for Vaccines and Immunization (Gavi) subsequent commitment to support its introduction, interest in economic evaluations assessing the cost-effectiveness, health impact, and potential cost savings associated with the use of TCV has increased among policymakers [24–26]. Despite this growing body of research, no recent review has been conducted to systematically organize and synthesize the evidence. A scoping review would help fill the gap by mapping the current research landscape on the economic implications of TCV, identifying knowledge gaps, and highlighting areas that future studies could focus on. This review aimed to map and summarize existing evidence on epidemiological models and economics of typhoid and TCV, including costs, cost-effectiveness analysis, and impact. It summarized findings from diverse epidemiological and economic analyses, covering different modeling approaches, vaccination strategies, treatment and immunization costs, public health outcomes, and cost-effectiveness results. The review focused on TCV given its benefits over other typhoid vaccines. Overall, it provides an overview of current evidence, identifies research gaps and offers insights to support decision-making in typhoid-endemic regions.

## Materials and methods

The scoping review followed the methodological framework outlined by Arksey and O’Malley [27], which organized the process into five sequential steps: (1) identification of the research question, (2) identification of relevant studies, (3) selection of eligible studies, (4) data extraction, and (5) collation, summarization, and reporting of the results. The review also followed the Preferred Reporting Items for Systematic Reviews and Meta-Analyses extension for scoping reviews (PRISMA-ScR) [28] to ensure transparency throughout the review process.

The review protocol was uploaded to the Open Science Framework (OSF): https://doi.org/10.17605/OSF.IO/WXDEU

### 1. Identification of the research question

This review addressed the research question: What evidence exists regarding the economic and public health impact of implementing the typhoid conjugate vaccine in a population?

To answer this question, the review focused on studies that reported outcomes such as cases averted, deaths averted, costs, reduction in incidence or transmission, life-years gained, or an incremental cost-effectiveness ratio (ICER). The research question helped guide the systematic search and selection of relevant studies.

### 2. Identification of relevant studies

An initial search was performed on PubMed using key terms such as “Costs,” “Cost-effectiveness,” “Model,” “Epidemiological Model,” and “Typhoid conjugate vaccine”. After reviewing the titles and abstracts obtained from the results of the initial search, relevant keywords were added to the search strategy to help refine it. A summarized version of the search strategy can be found in Table 1, with the detailed search strategy outlined in the S1 Appendix.

**Table 1:** Search strategy.

**#1**	Cost* OR Cost-effectiveness OR Cost effective OR Cost-effective* OR Economic evaluation OR Cost-benefit OR Cost benefit OR Cost-utility OR Cost utility OR Cost analys* OR Cost-analys*
**#2**	Model* OR Dynamic model OR Mathematical model OR Population model OR Epidemiological model OR Stochastic model OR Static model OR Transmission model
**#3**	Typhoid vaccin* OR Typhoid conjugate vaccine OR TCV
**Final query**	#1 OR #2 AND #3

The refined search strategy was then used by two authors (P.M. and J.K.) to search across several databases that included PubMed, Web of Science, International Health Technology Assessment (HTA), Scopus, and the National Health Service Economic Evaluation Database (NHS EED). Reference lists of all the included studies were screened, and Google Scholar was used to help identify additional literature. The final database search was done on January 20, 2025.

### 3. Selection of eligible studies

The results from the database searches were imported into Covidence [29] for duplicate removal, title and abstract screening, and full-text review. Two reviewers (P.M. and J.K.) independently screened all the titles and abstracts using the inclusion and exclusion criteria listed in Table 2. Screening happened in two stages. First, titles and abstracts were checked to flag relevant studies. If a title looked relevant but the abstract didn’t provide enough detail, the study was carried forward for full-text review. The next stage involved reviewing the full texts to help decide whether the study should be included in the review based on the inclusion criteria. For any studies that were excluded at this stage, the reason for exclusion was recorded to maintain transparency.

**Table 2:** Inclusion criteria.

Criteria	Inclusion	Exclusion
**Language**	English	Non-English
**Publication Type**	Peer-reviewed articles, full text available	Grey literature, full text unavailable
**Model requirement**	Model parameters must directly include TCV	Models lacking TCV-specific parameters
**Study Methodology**	Quantitative studies with empirical data	Qualitative studies, Opinion pieces, editorials, literature reviews, books
**Outcomes**	- Cost of illness of typhoid - Cost of vaccine delivery/vaccination for TCV - Public health impact of TCV - Cost-effectiveness of TCV	Studies without these specific outcomes

**Fig 1:**
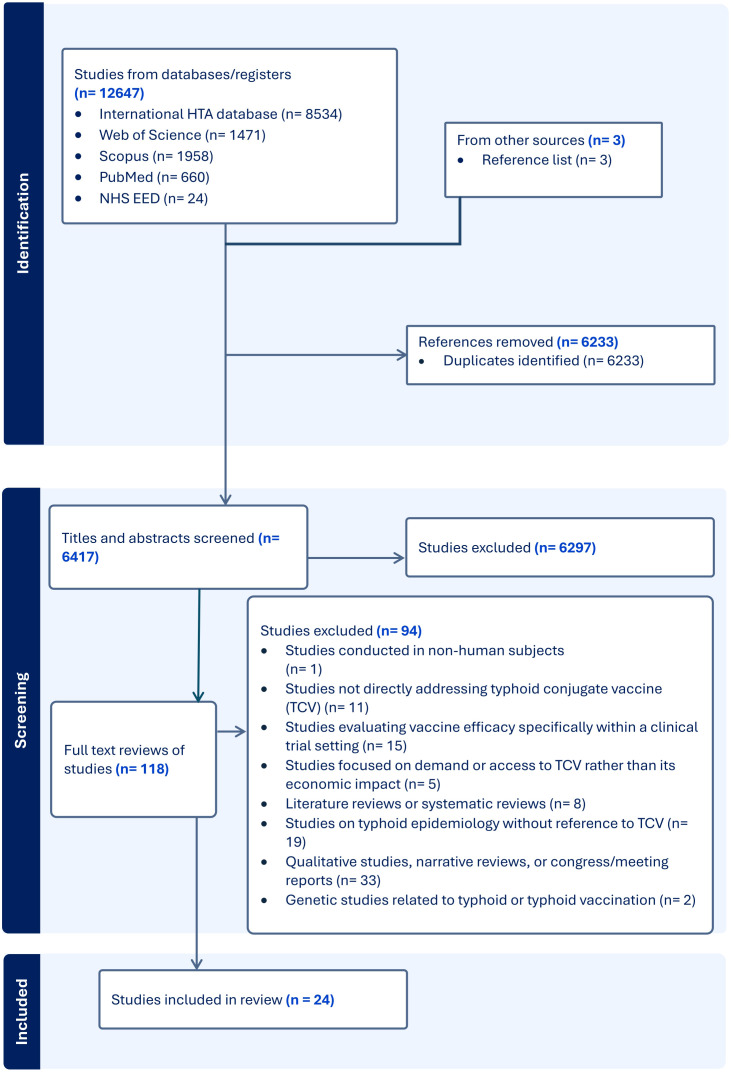
Economic and public health impact PRISMA flow diagram. Flow diagram illustrating the number of records identified, screened, included, and excluded during the study.

Study selection followed the PRISMA-ScR guidelines [28] (S2 Appendix), and the process is outlined in a PRISMA flow diagram (Fig 1) to ensure transparency and reproducibility. If the two reviewers (P.M. and J.K.) disagreed at any point, a third reviewer (S.O.) was consulted to help resolve the differences and reach a consensus.

### 4. Data extraction

Data were extracted from the 26 included studies using a Microsoft Excel template (S3 Appendix) to ensure accuracy and completeness. Key details from each study were extracted, including the title, lead author, year of publication, vaccination strategies, model type, time horizon, perspective, cycle length, and parameters included in the epidemiological model. The template also captured key outcomes such as the cost of treatment, vaccination costs, public health outcomes, ICERs, and other major findings.

### 5. Collation, summarization, and reporting of results

The analysis provided a descriptive summary of the quantitative data and sorted the included studies into four sub-groups: i) cost of illness, ii) cost of vaccination, iii) public health impact, and iv) economic evaluation. Results for each sub-group were organized and presented in separate tables to help highlight patterns and trends.

### Cost adjustment over time

To allow for comparison across studies, all monetary values were adjusted to 2024 USD. Values originally reported in USD were first converted to their equivalent local currency unit (LCU) for each country based on the average annual exchange rate for the base year of the reported cost data. The resulting LCU value was then inflated to the equivalent 2024 value, using country specific gross domestic product (GDP) deflators. The resulting 2024 LCU values were then converted back to USD values using the country specific annual exchange rate for 2024. All country specific annual average exchange rates and GDP deflator values were obtained from the World Bank Group [30,31]. When data for specific years were missing from the World Bank Group sources, alternative values were obtained from data reported by the respective country’s central bank or other relevant literature [32–35]. For the study that reported monetary values in 2015 international dollars [36], we directly adjusted these to 2024 USD using the consumer price index [37]. This assumed that 1 international dollar is equivalent to 1 USD, which is consistent with The World Bank’s definition [38]. We used the same approach for the study by Lo et al., which was conducted in typhoid-endemic regions but did not specify the countries included in the analysis [39].

### Quality assessment

All economic evaluations included in this review were assessed using the Drummond 10-point checklist [40] (S4 Appendix), which is a tool used for evaluating economic evaluations of health interventions. The process involved two reviewers (P.M. and J.K.) independently scoring each study by awarding one point for each criterion met. The total score was computed and an overall rating was provided based on the Doran’s classification system [41], which rated studies as poor (total score: 1–3), average (total score: 4–7), or good quality (total score: 8–10).

For studies focusing solely on the public health impact of TCV, a tool that assessed infectious disease models was considered [42]. However, this tool mainly focused on aspects of the software used and data availability, which made it unsuitable for our review.

No standardized quality assessment tool was identified for cost of illness or cost of vaccination/ vaccine delivery studies. We therefore assumed that studies published in peer-reviewed journals were of acceptable quality. This approach helped maintain consistency across the different types of studies included in this review.

All included economic evaluations were rated as “Good” quality, with full details shown in S5 Appendix.

## Results

### General characteristics of the included studies

26 studies published between 1998 and 2024 were included in the review, with 6 studies being multi-country analyses. Across the 26 studies, the number of distinct countries represented per region were: Sub-Saharan Africa (36 countries), South Asia (8 countries), East Asia and the Pacific (13 countries), Europe and Central Asia (8 countries), Latin America and the Caribbean (6 countries), and the Middle East and North Africa (4 countries). Two studies looked at LMICs where typhoid is endemic [39,43].

Of the 26 studies, ten assessed the COI for typhoid [44–53], four estimated the cost of TCV vaccination [54–57], one estimated the cost of TCV delivery [58], two modeled public health impact [43,59] and nine conducted economic evaluations [25,36,39,60–65]. This information is summarized and presented in Table 3.

**Table 3:** Summary of included studies.

Study Type	Country/Region	Number of studies	Key Outcomes Reported	References
Cost-of-illness	Bangladesh, China, India, Indonesia, Malawi, Nepal, Pakistan, the Republic of Nauru, Tanzania, and Vietnam.	10	Outpatient costs, inpatient costs, direct medical costs, direct non-medical costs, indirect costs	[44–53]
Cost-of-Vaccination/Delivery	India, Malawi, and Zimbabwe	5	Total vaccination cost and vaccine delivery costs in terms of financial and economic costs	[54–58]
Modelling studies: Public health impact	Endemic areas, Afghanistan, Angola, Armenia, Azerbaijan, Bangladesh, Benin, Bhutan, Bolivia, Burkina Faso, Burundi, Cambodia, Cameroon, Central African Republic, Chad, Comoros, Congo Rep, Côte d’Ivoire, Cuba, DR Congo, Djibouti, Eritrea, Ethiopia, Gambia, Georgia, Ghana, Guinea, Guinea Bissau, Guyana, Haiti, Honduras, India, Indonesia, Kenya, Kiribati, DPR Korea, Kyrgyz Republic, Lao PDR, Lesotho, Liberia, Madagascar, Malawi, Mali, Mauritania, Moldova, Mongolia, Mozambique, Myanmar, Nepal, Nicaragua, Niger, Nigeria, Pakistan, Papua New Guinea, Rwanda, São Tomé e Príncipe, Senegal, Sierra Leone, Solomon Islands, Somalia, Sri Lanka, Republic of Sudan, South Sudan, Tajikistan, Tanzania, Timor Leste, Togo, Uganda, Ukraine, Uzbekistan, Vietnam, Yemen, Zambia, and Zimbabwe.	2	Percentage reduction in cases, Cases averted, Deaths averted, antimicrobial-resistant cases averted	[43,59]
Economic evaluations	Afghanistan, Bangladesh, Benin, Burkina Faso, Burundi, Cambodia, Cameroon, Central African Republic, Chad, Comoros, DR Congo, Côte d’Ivoire, Djibouti, Eritrea, Ethiopia, Gambia, Ghana, Guinea, Guinea-Bissau, Haiti, India, Kenya, North Korea, Kyrgyzstan, Lao PDR, Lesotho, Liberia, Madagascar, Malawi, Mali, Mauritania, Mozambique, Myanmar, Nepal, Nicaragua, Niger, Nigeria, Pakistan, Papua New Guinea, Rwanda, São Tomé and Príncipe, Senegal, Sierra Leone, Solomon Islands, Somalia, South Sudan, Sudan, Tajikistan, Tanzania, Togo, Uganda, Vietnam, Yemen, Zambia, Zimbabwe, and LMICs	9	Life-years gained, Quality adjusted life years gained (QALY), disability adjusted life years averted (DALY), ICER	[25,36,39,60–65]

S1 Table (Supporting information) provides a comprehensive overview of all extracted studies, capturing detailed unadjusted cost values from each study’s findings before similar findings were grouped.

### Cost of illness

Ten of the included studies [44–53] examined the economic burden of typhoid fever. The studies were summarized based on the location, perspective, population, cost parameters, and key findings. The variation in outcomes between the studies were attributed to the type of costs included (direct medical, non-medical, and/or indirect), region, patient demographics, and healthcare setting.

Four studies used a single perspective, either societal [44,50,51] or patient [47], while six studies adopted multiple perspectives, with four combining healthcare provider and patient perspectives [45,46,52,53] and two combining healthcare provider and societal perspectives [48,49]. In terms of the study population, seven studies included patients of all ages [44–47,49,52,53], one focused on those older than 2 months [51], another on those over 6 months [50], and one varied the age group by country [48].

The reported outpatient costs varied widely, ranging from USD 1.3 to an estimated USD 337.1 [45,46,48,49,52,53], with significant differences across regions. In Sub-Saharan Africa, the reported outpatient cost was USD 34.8 [45]. In South Asia, it ranged from USD 1.3 to USD 159.1 [46,48,52,53], and in East Asia and the Pacific, costs were higher (USD 101.1 to USD 337.1) [48,49]. Inpatient treatment costs were higher than outpatient costs, ranging from USD 72.4 to USD 2028.0 [44–51]. Regional comparisons of inpatient costs showed a similar trend, with Sub-Saharan Africa (USD 153.8 to USD 307.2) [45,51] and South Asia (USD 72.4 to USD 316) [44,46,48,50,52,53] reporting lower costs when compared to East Asia and the Pacific (USD 417.6 to USD 2028.0) [47–49]. Inter-regional differences in East Asia and the Pacific were further highlighted by Poulos et al. [48], where the inpatient cost in Vietnam was USD 417.6 compared to USD 845 in Indonesia.

The direct medical costs (e.g., medication costs, laboratory and imaging costs, bed costs, consultation fees, medical procedures, and treatment costs for relapses/complications) varied by age and type of care (outpatient/ inpatient). Outpatient direct medical costs ranged from USD 1.0 to USD 133.2 [45,46,52,53]. When comparing age groups, children under 15 years had reported costs of USD 1.0 to USD 34.8 [45], while adults had higher direct medical costs of USD 1.0 to USD 133.2 [45,46,52,53]. The direct medical costs for inpatients ranged from USD 1.8 to USD 1789 [44–47,51–53], with lower estimates reported for children (USD 2.0 to USD 38.1) [45], when compared to adults (USD 1.8 to USD 1789) [44,46,47,51–53].

Direct non-medical costs included transportation, meals, accommodation, lodging, paid care services, incidental payments, and other household expenditures. These costs were higher for inpatients (USD 3.8 to USD 54.8) [44–46,51–53] than outpatients (USD 1.0 to USD 5.0) [45,46,52,53].

The economic burden of typhoid fever goes well beyond direct medical costs. Indirect costs as a result of income lost due to missed work were substantial, especially for hospitalized patients. This was highlighted by the included studies that reported the indirect costs for outpatients to range from USD 8.6 to USD 26.8 [45,46,52,53], while those for inpatients ranged from USD 27.4 to USD 920 [44–47,50–53]. Some studies found that the indirect costs incurred by inpatients even exceeded their direct medical costs [45,51].

Complications of typhoid fever further increase the financial burden. Three studies [46,49,50] explored the inpatient costs associated with treating these complications. Punjabi [49], estimated costs of treating severe cases to exceed USD 560, while Mejia et al. [46] reported the cost of treating intestinal perforation of USD 514.2. Finally, Gupta et al. [50] reported the cost of managing sequelae, death, or referred cases as USD 127.8. The detailed findings are presented in Table 4.

**Table 4:** Summary of cost of typhoid illness studies.

Country, Year (Reference)	Perspective	Study Population	Cost of illness parameters	Key Findings (USD, 2024)					
India, 2021 [44]	Societal	1 275 hospitalized patients with blood culture–confirmed enteric fever or ileal perforation at 14 hospitals **(All ages)**	**Direct medical cost:** User charges, diagnostic charges, and procedure/surgery **Direct non-medical costs:** Travel, meal, lodging, and other costs **Indirect cost:** Income lost by a patient and/or caretaker, and payment to a substitute						
					Tier 2*	Tier 3*			
				**Direct Medical Costs**	$108.0	$362.0			
				**Direct Non-Medical Costs**	$19.6	$52.4			
				**Indirect Costs**	$72.4	$172.7			
Malawi, 2022 [45]	Healthcare provider & patient	109 lab-confirmed typhoid cases from two primary and one referral facility **(All ages)**	**Direct medical cost:** Consultation, staff time, drugs, and investigation **Direct non-medical costs:** Travel costs, hotel, meal costs for patient and/or parent and/or visitor **Indirect cost:** Self-reported income lost by a parent/caregiver for children or self-reported lost earnings by adult patient.	**Household perspective**					
					**Children (<15y)**	**Adults**			
				**Mean Direct Medical Costs**					
				Inpatient	$2.0	$1.8			
				Outpatient	$1.0	$1.0			
				**Mean Direct Non-Medical Costs**					
				Inpatient	$24.0	$54.8			
				Outpatient	$1.0	$1.0			
				**Mean Indirect Costs**					
				Inpatient	$38.5	$56.8			
				Outpatient	$24.0	$9.9			
				**Healthcare provider perspective**					
					**Children (<15y)**	**Adults**			
				**Total cost:**					
				Inpatient	$196.5	$307.2			
				Outpatient	$34.8	$32.2			
Pakistan, 2020 [46]	Healthcare provider & patient	1029 blood culture confirmed enteric fever or ileal perforation cases in four Karachi hospitals **(All ages)**	**Direct medical costs:** Registration, examination, inpatient stay, laboratory tests, drugs, and other services **Direct Nonmedical costs:** Transport, Food, Lodging, and Childcare **Indirect costs for patient** Days spent seeking care, Days unable to work, Days of sick leave, School days lost **Indirect costs for caregiver:** Days spent with patient, days unable to work, days of sick leave	**Patient perspective** **Median direct medical cost** Inpatient: $196.9 Outpatient: $40.0 **Median direct nonmedical** Inpatient: $54.5 Outpatient: $3.3 **Median productivity loss** Inpatient: $25.4 Outpatient: $19.4 **Median cost of illness** Inpatient: $263.8 Outpatient: $45.2 **Healthcare provider perspective** **Outpatient** Hospital A: $4.4 Hospital B: $21.4 **Weighted mean cost per case** Hospital A: $51.5 Hospital B: $52.8 Hospital C: $11.9 **NB:** Hospital A (Public) and B + C (non-profit)					
The Republic Of Nauru, 2001 [47]	Patient	50 patients **(All ages)**	**Direct medical costs:** Physician fee, laboratory, hospitalization, and Medication costs **Indirect costs for the patient** Loss of income	**Patient perspective** **Mean total direct cost per patient:** $1789 **Mean total indirect cost per patient:** $920 **Mean total cost:** $2028					
China, India, Indonesia, Pakistan and Vietnam, 2011 [48]	Healthcare provider and Societal	China: **5–60 years** India: **all ages** Indonesia: **all ages** Pakistan: **2–15 years** Vietnam: **5–18 years**	**Direct costs** Outpatient charges, diagnostic tests. medicine, Examination, Bed charges **Nonmedical items** Meals, lodging, and transport **Indirect costs** Patient’s, substitute laborers,” caretakers’ and other persons’ lost income/production	**Healthcare provider perspective**					
					**Hue**	**Hei chi**	**North J**	**Karachi**	**Kolkata**
				**Mean total cost**					
				Inpatient	$417.6	$425	$848	$280.5	$195
				Outpatient	$101.1	$132.4	$131.5	$76.1	$19.7
				**NB:** North J = North Jakarta					
Indonesia, 1998 [49]	Healthcare provider and Societal	Modeled national burden based on incidence 358–810/100 000 (200 M population) **(All ages)**	**Direct costs:** Medication, surgery, bed charges, consultation **Indirect costs:** Loss of work for patient/caregiver	**Healthcare provider perspective** **Mean estimated treatment cost** Inpatient (w/o complications): $224.7 - $1123.6 Inpatient (with complications): > $561.8 Outpatient: $112.0 - $337.1					
India, 2024 [50]	Societal	1 650 hospitalized fever cases **(>6 months)**	**Direct Medical costs:** Medication, laboratory tests, bed charges **Direct Non-medical costs** **Indirect costs**	**Patient perspective**					
					**(<15y)**	**Adults**	**No comp.**	**Comp.**	
				**Direct cost**	$55.8	$57.3	$52.2	$86.3	
				**Indirect cost**	$16.6	$53.6	$46.0	$41.5	
				**Total treatment cost**	$72.4	$110.8	$98.1	$127.8	
				**NB:** Comp. = Complications					
Tanzania, 2014 [51]	Societal	17 blood culture–confirmed typhoid episodes at three Pemba district hospitals **(>2 months)**	**Direct cost:** Treatment expenditure Travel expenditure Meal expenditure (before, during, and after hospital care) **Indirect cost:** Productivity loss (patient’s absence from work) Productivity loss (caregiver’s absence from work)	**Societal perspective**					
					**Children (<15y)**	**Adults**			
				**Direct cost**	$38.11	$38.11			
				**Indirect cost**	$172.4	$128.5			
				**Total treatment cost**	$210.5	$153.8			
				**NB:** Direct costs were not disaggregated by age, so the same cost was entered above for adults and children					
Bangladesh 2020 [52]	Healthcare provider & patient	1772 patients aged under 18 years	**Direct medical costs** Registration, Clinical examination, inpatient stay, laboratory tests, medication, other services **Direct Nonmedical costs** Transport, food, lodging, childcare **Indirect costs for patient** Days spent seeking care, days unable to work, days of sick leave, school days lost **Indirect costs for caregiver** Days spent with patient, days unable to work, days of sick leave	**Patient perspective** **Median direct medical cost:** Inpatient: $189.1 Outpatient: $38.4 **Median direct nonmedical cost was** Inpatient: $52.4 Outpatient: $3.1 **Median productivity loss:** Inpatient: $24.4 Outpatient: $18.7 **Median cost of illness** Inpatient: $253.4 Outpatient: $43.4 **Healthcare provider perspective (Enteric Fever)** **Outpatient** Hospital A: $12.5 Hospital B: $1.3 **Mean cost per case** Hospital A: $73.27 Hospital B: $36.2 Weighted cost: $57.0 **NB:** Hospital A and B (Private non-profit)					
Nepal 2020 [53]	Healthcare provider & patient	395 patients of all ages	**Direct medical costs** Registration, Clinical examination, inpatient stay, laboratory tests, medication, other services **Direct Nonmedical costs** Transport, food, lodging, childcare **Indirect costs for patient** Days spent seeking care, days unable to work, days of sick leave, school days lost **Indirect costs for caregiver** Days spent with patient, days unable to work, days of sick leave	**Patient perspective:** **Median direct medical cost:** Inpatient: $153.8 Outpatient: $29.6 **Median direct nonmedical cost was** Inpatient: $18.2 Outpatient: $3.8 **Median productivity loss:** Inpatient: $37.4 Outpatient: $8.6 **Median cost of illness** Inpatient: $209.2 Outpatient: $42.9 **Healthcare provider perspective (Enteric fever)** **Outpatient** Hospital A: $12.6 Hospital B: $8.5 **Mean cost per case** Hospital A: $103.1 Hospital B: $79.0 Weighted cost: $88.1 **NB:** Hospital A and B (Teaching hospital)					

*Tier 2: smaller hospitals in five rural and one urban site; Tier 3: eight tertiary care hospitals in India

### Cost of vaccine delivery/ cost of vaccination of TCV

We identified four cost of vaccination studies [54–57] and one cost of vaccine delivery study [58]. Four of the five studies reported vaccine coverage based on primary data, with coverage ranging from 46.5% to 76% in India [54,57], 77% in Malawi [58], and 66.7% to 97.1% in Zimbabwe [56]. In contrast, Debellut et al. [55] used estimates for vaccine coverage rates ranging from 80% to 95%, depending on the strategy and year. In terms of vaccine delivery strategies, India conducted campaigns targeting children aged 9 months to 15 years [54,57], while Malawi used both routine immunization for children >9 months alongside campaigns for children aged 9 months to 15 years [55,58]. Zimbabwe, reported on a campaign that targeted a wider age group of 6 months to 45 years [56].

The four cost of vaccination studies [54–57] incorporated costs associated with program-level activities (planning and preparation, microplanning, training, sensitization, social mobilization, supervision and monitoring, and AEFI preparedness and management), as well as costs directly attributed to the vaccine procurement (vaccine cost, syringe cost, and safety box cost). The cost of vaccine delivery study from Malawi [58] focused only on the cost of delivering the vaccine and excluded the procurement cost of vaccines, syringes, safety boxes, and supplements.

Studies reporting both financial and economic costs [54,55,57,58] found that economic costs consistently exceeded financial costs. Economic costs captured the full value of resources used, including the market value of donated or co-financed vaccines and supplies, and opportunity costs such as staff time, shared facilities, and capital equipment, while financial costs only included actual expenditures incurred by the country. Regional differences were seen in co-financing mechanisms, with India receiving vaccines through donations from the manufacturer [54,57], while Malawi and Zimbabwe, received support from Gavi to cover part of their operational and vaccine costs [55,56,58]. Economic costs for vaccination in India were more than three times the financial costs for routine vaccination and nearly two times higher for campaigns [54,57]. In Malawi, the economic cost of vaccination through routine vaccination was four times greater than the financial cost, while it was twice as high for campaigns [55,58].

Among the five studies, three were retrospective empirical analyses [54,56,57], one projected the costs [55], and one combined empirical data for immunization through campaigns with projected costs for routine delivery [58]. Of the four cost of vaccination studies [54–57], only one study estimated the total cost of routine vaccination [55], while all reported the total cost for vaccination through campaigns [54–57]. For vaccine delivery (excluding procurement costs), campaign delivery costs ranged from USD 0.8 to USD 1.6 (economic), while routine delivery ranged from USD 0.7 to USD 2.4 (economic) [54,55,57,58]. The economic cost of vaccine delivery through campaigns in India was double that reported for Malawi [54,55,57,58]. Table 5 summarizes the cost of vaccination and vaccine delivery studies for TCV.

**Table 5:** Summary of cost of vaccination and vaccine delivery studies for TCV.

Country, Year (Reference)	Strategies	Vaccination parameters	Source of costs	Key Findings (USD, 2024)
India, 2020 [54]	**Campaign:** 9 months to 14 years	**Vaccine related costs:** Cost of vaccine Cost of Syringe Cost of Safety Box Vaccine wastage: 3% Vial size: 5 doses Vaccine coverage = 46.5%-76.1% **Vaccination activities:** Planning & preparation Sensitization Training Microplanning Social mobilization Service delivery Adverse event following immunization (AEFI) management. Supervision Monitoring	Navi Mumbai Municipal Corporation officials, the National Technical Advisory Group on immunization and other key stakeholders	**Vaccine coverage** 46.5%-76.1% **Cost of vaccination per dose:** **Campaign** $4.6 (Financial) $8.0 (Economic). **Cost of delivery only per dose:** **Campaign** $0.5 (Financial) $1.5 (Economic)
Malawi, 2022 [55]	**Routine:** > 9 months **Campaign:** > 9 months to <15years	**Vaccine related costs:** Cost of vaccine Cost of Syringe Cost of Safety Box Vaccine & syringe wastage: 10% Safety box wastage: 5% Vial size: 5 doses Vaccine procurement additional costs (% of vaccine price) 21% Supplies procurement additional costs (% of supply price) 31% **Estimated coverage:** **Initial:** 95% in campaign **Routine:** 2023, 80% 2024, 84%	Primary data collection, Gavi and UNICEF country office	**Estimated vaccine coverage:** 95% (Campaign 2022) 80% (Routine 2023) 84% (Routine 2024) **Cost of Vaccination per dose:** **Routine** $0.8 (Financial), $2.4 (Economic) **Campaign** $0.7 (Financial), $2.6 (Economic) **Cost of delivery only per dose:** **Routine** $0.2 (Financial), $0.7 (Economic) **Campaign** $0.4 (Financial), $0.8 (Economic)
Zimbabwe, 2022 [56]	**Campaign:** > 6 months to <45 years	**Vaccine related costs:** Cost of vaccine Cost of Supplies Vaccine wastage: < 0.01% Vaccine coverage = 66.7%-97.1% **Vaccination activities:** Planning & preparation Sensitization Training Microplanning Social mobilization Service delivery Adverse event following immunization (AEFI) management. Supervision Monitoring	Surveillance data, WHO, UNICEF, Ministry of Health and Child Care, Harare City Health Department,	**Vaccine coverage:** 85.4% (Campaign) **Cost of vaccination per dose:** **Campaign** $16.00 (Financial) **Cost of delivery only per dose:** **Campaign** $5.3 (Financial)
India, 2023 [57]	**Campaign:** > 9 months - < 15 years	**Vaccines costs:** Vaccine cost Syringe cost Safety box cost Vaccine coverage achieved 71.0% Vaccine wastage 3.1% Syringe wastage 4.5% **Activities:** Planning and Preparation Microplanning Training Sensitization Social Mobilization Service Delivery Supervision and Monitoring AEFI Preparedness and Management	Navi Mumbai Municipal Corporation officials, the National Technical Advisory Group on immunization and other key stakeholders	**Vaccine Coverage:** 71% (Campaign) **Cost of Vaccination per dose:** **Campaign** $2.4 (Financial) $4.74 (Economic) **Cost of delivery only:** **Campaign** $0.4 (Financial) $1.6 (Economic)
Malawi, 2024 [58]	**Routine:** > 9 months **Campaign:** > 9 months to <15years	**Program activities financial costs:** Estimating demand for routine (Meetings) Training (Fuel, maintenance & energy) Management (Transport) Social Mobilization (Stationary & communication) Vaccine distribution & storage (Per diem) Vaccine service delivery (Supplies) **Program activities opportunity costs:** Estimating demand for routine (Human resource) Training (capital costs of vehicles & equipment) **Others** Supervision Waste management AEFI management (Campaign) Record keeping & Monitoring Vaccine coverage = 77%	Primary data collection using questionnaires from health facilities, EPI offices and vaccine stores. Secondary data used from different sources	**Vaccine Coverage:** 77% (Campaign) **Projected cost of vaccine delivery only per dose:** **Routine** $0.4 (Financial) $2.4 (Economic) **Campaign** $0.5 (Financial) $0.8 (Economic)

### Public health impact studies

Two studies [43,59], evaluated the public health impact of different typhoid vaccination strategies for TCV. Both studies used a 10-year time horizon [43,59] and employed a dynamic transmission model. These models incorporated parameters for demographics (birth and death rates), disease (infection duration, immunity duration, and carrier probability), and transmission dynamics (basic reproductive number, proportion symptomatic, and chronic carrier infectiousness), along with vaccine characteristics (initial efficacy, duration of protection, and waning immunity), and antimicrobial resistance dynamics (resistance acquisition rates, transmission risks, and recovery rates). However, as no published study had reported real-world data to quantify the protection conferred by TCV through herd immunity prior to 2021 [15], neither of the studies incorporated it as a model parameter. The models included key infection states such as susceptible, infected, recovered, vaccinated, and chronic carrier.

Birger et al. [59] modeled a combination of routine vaccination at 9 months and campaign vaccination strategies targeting children aged 9 months to 15 years across 73 Gavi-eligible countries. In contrast, Kaufhold et al. focused on endemic countries, analyzing routine immunization at 9 months under varying coverage levels that ranged from no vaccination up to 100% coverage [43]. Higher coverage consistently led to more typhoid cases being averted. Kaufhold et al [43], projected a 44% reduction in cases at 80% vaccination coverage and 58% reduction in cases at 100% coverage. A proportional decrease in AMR cases was also observed. Similarly, Birger et al. [59] reported that for every 1% increase in vaccine coverage, the projected number of cases averted rose by 0.3%.

Both studies conducted a sensitivity analysis and identified influential parameters. Kaufhold et al [43] found transmission rates, symptomatic proportions among vaccinated individuals, and chronic carrier infectiousness as sensitive parameters, whereas Birger et al. [59] identified population age distribution and vaccine coverage as the most influential parameters, with younger populations showing greater case reduction. A summary of the findings from the public health impact studies are presented in Table 6

**Table 6:** Summary of public health impact (modelling) of TCV studies.

Country, Year (Reference)	Strategy	Model Type	Model Parameters	Key Findings
Afghanistan, Angola, Armenia, Azerbaijan, Bangladesh, Benin, Bhutan, Bolivia, Burkina Faso, Burundi, Cambodia, Cameroon, Central African Republic, Chad, Comoros, Congo Rep, Côte d’Ivoire, Cuba, DR Congo, Djibouti, Eritrea, Ethiopia, Gambia, Georgia, Ghana, Guinea, Guinea Bissau, Guyana, Haiti, Honduras, India, Indonesia, Kenya, Kiribati, DPR Korea, Kyrgyz Republic, Lao PDR, Lesotho, Liberia, Madagascar, Malawi, Mali, Mauritania, Moldova, Mongolia, Mozambique, Myanmar, Nepal, Nicaragua, Niger, Nigeria, Pakistan, Papua New Guinea, Rwanda, São Tomé e Príncipe, Senegal, Sierra Leone, Solomon Islands, Somalia, Sri Lanka, Republic of Sudan, South Sudan, Tajikistan, Tanzania, Timor Leste, Togo, Uganda, Ukraine, Uzbekistan, Vietnam, Yemen, Zambia and Zimbabwe, 2022 [59]	**Routine + Campaign:** > 9 months to <15years **Time Horizon:** 10 years	**Dynamic transmission model** S1, S2, + I1, S, I1,R, I2,S, I2,R, IT, + V1, V2, + CS, CR, + R.	**Demographic parameters** Crude birth rate (per year) (µ) Crude death rate (per year) (µ) **Fixed disease parameters** Mean duration of infectiousness (1/δ) 4 weeks Fraction infected who become carriers (θa) Duration of immunity (1/ω) 104 weeks **Other Parameters** Basic reproductive number 1·5, 2·5, 3·5, 7, 10·5, Proportion symptomatic 0·01, 0·05, 0·10, 0·5, Vaccination coverage 70–79%, 62–80%, 86–95%. Relative infectiousness of chronic carriers (r) **Vaccine-related parameters** Initial efficacy of TCV (ν) 87.5%, Uniform(80%, 95%) Waning of vaccine-induced immunity (ωV) 0·0672 per year, Gamma (1·40, 0·0479) **Resistance parameters** Rate of resistance acquisition from treatment 0·01–5 per week, Relative risk of transmission for resistant strain 0·3–3, Rate of recovery from primary infection with a resistant strain 0·075–0·7 per week, Rate of recovery from subclinical infection with a resistant strain 0·075–0·7 per week.	**General** - Predicted cases averted 66.7 million (48·1–88·3 million) cases, i.e., 46–74%. - Predicted deaths averted 826000 (381000- 2.4 million) deaths. - Predicted DALYs averted 44.4 million (18.7-143 million) DALYs. **Anti-microbial resistant typhoid** - Fluoroquinolone non-susceptibility (FQNS) cases averted 42·5 million (24·8–62·9 million) cases. - Multidrug resistance cases averted 21·2 million (16·4–26·5 million) cases - Overall antimicrobial-resistant typhoid fever averted 53·5 million (37·3–75·3 million) cases - Predicted reduction of resistant cases proportion 16.1% (0–49%) - Prevalence of resistant cases expected to decrease by 1.1% for every one-unit increase of R0. **Influential parameters** Population age distribution and vaccine coverage.
Endemic areas, 2019 [43]	**Routine** only at 9 months **Time Horizon:** 10 years **Scenarios:** no vaccination, 30% coverage, 50% coverage, 80% coverage, and 100% coverage	**Dynamic transmission model** S1, S2, + I1, S, I1,R, I2,S, I2,R, IT + V + CS, CR, + R.	**Demographic parameters:** Birth and death rate **Fixed disease parameters:** Duration of infectiousness for first antimicrobial-sensitive inf.: 4 weeks Fraction who experience disease-induced mortality Duration of temporary immunity following recovery from natural infection, 104 weeks **Other parameters:** Transmission parameter 0.3 (0.05–0.5) Vaccine efficacy (initial) 0.95 (0.8–1) Reduction in risk of infection for vaccinated 1 (0.5–1) Duration of protection from vaccination 19.2 years Fraction symptomatic 0.2 (0.1–1), Fraction symptomatic for vaccinated individuals 0.05 Fraction treated 0.75 (0–1) Duration of infectiousness with treatment 1 week, Relative infectiousness of chronic carriers 0.35 Fraction that become carriers from 1st, antimicrobial-sensitive infection 0.03 Relative rate of recovery from second infection 1 **Resistance parameters** Rate of treatment-induced/acquired resistance Natural recovery rate from first, antimicrobial-resistant infection 0.2 (0.1–0.5)/ week Relative infectiousness of resistant strain(s) 0.9 Fraction becomes carriers from the 1st, AMR infection	**No Vaccination Group:** 483 cases per 100,000 person-years. **80% Coverage:** Reduced to 270 cases per 100,000 person-years, a 44% decrease. **100% Coverage:** Further reduced to 202 cases per 100,000 person-years, representing the maximum reduction of 58%. **Influential parameters** Transmission rate and proportion of cases among vaccinated individuals that are symptomatic, followed by the duration of immunity afforded by vaccination, and the relative infectiousness of chronic carriers.

### Economic evaluations (cost-effectiveness analysis)

There were nine cost-effectiveness analyses [25,36,39,60–65] included in this review. Seven utilized a 10 year time horizon [25,36,39,61,62,64,65], one employed a 15-year horizon [60], and one a life-time horizon [63]. The studies used models that incorporated demographic, disease transmission, and vaccine-related parameters. All studies conducted sensitivity analyses to identify key drivers of the cost-effectiveness related outcomes.

The vaccination strategies were compared to a baseline “do-nothing” scenario to estimate the incremental benefits of TCV. Seven studies assessed both routine vaccination and campaigns [25,36,39,61,62,64,65], one analyzed campaign-only vaccination [63], and one varied vaccine efficacy in routine immunization to simulate different vaccine strategies [60].

Case reductions for routine vaccination ranged from 2% to 84%, while for routine vaccination with a campaign ranged from 6% to 95% [25,36,39,60,62,64,65]. Deaths averted ranged from 0% to 36% for routine-only vaccination in low-incidence areas, to 17% to 100% when combined with campaigns [25,36,60].

Across studies, the reported ICER varied based on the type of health outcome and typhoid vaccination strategy used. Health outcomes were primarily measured in DALYs [25,36,39,61,62,64,65], with two studies opting to use quality-adjusted life years (QALYs) [60,63]. TCV vaccination strategies were consistently found to be cost-effective compared to no vaccination, with the exception of the analysis in rural settings by Chauhan et al. [60] and the study conducted in Lao PDR [63], where low disease incidence rendered no vaccination the most optimal choice. Antillón et al. [36], Lo et al. [39] and Burrows et al. [65], identified routine-only vaccination as the optimal choice under specific scenarios, including higher vaccine prices [36], level of incidence (40–75 cases per 100,000) [39] and specific modelling assumptions (Model A) [65]. Conversely, these same studies reported routine vaccination with catchup-campaigns to have the lowest ICERs under conditions of lower vaccine prices [36], high incidence (>130 cases per 100,000) [39] and when utilizing models capturing broader transmission benefits (Models B and C) [65]. Chauhan et al. [60] also found combined vaccination strategies to be the optimal choice in urban settings. Four studies [25,61,62,64] concluded that combining routine vaccination with campaigns was more cost-effective, with routine-only strategies often dominated. Amongst these four studies, Weyant et al. [64] used targeted campaigns that included schools, and Ryckman et al. [62] included both community and school-based campaigns.

Studies adopting a societal perspective (which included direct medical, direct non-medical, and indirect costs) [39,60,62,64,65] consistently reported lower ICERs than those using a healthcare provider perspective (only included direct medical costs). This was highlighted by Chauhan et al.,reporting substantially lower ICER values from the societal perspective compared to the healthcare provider perspective, showing TCV to be cost saving when broader societal costs were included [60]. Similarly, two studies that evaluated cost-effectiveness using DALYs reported lower ICERs from the societal perspective than from the healthcare provider perspective [62,64].

High-incidence areas consistently demonstrated greater cost-effectiveness for TCV compared to low-incidence areas. This was highlighted by five studies [25,36,39,60,62] which reported that in high-incidence settings, TCV was not only cost-effective but also cost-saving, offering greater health benefits at lower costs than alternative strategies. These studies are summarized in Table 7.

**Table 7:** Summary of economic evaluations of TCV.

Country, Year (Reference)	Vaccination Strategy	Model Type	Costs Reported	Sensitive Parameters	Key Findings																		
India, Kenya and Vietnam, 2017 [36]	**Base:** Do nothing **Comp 1:** Routine vaccination at 9 months **Comp 2:** Routine + Catch-up <5years **Comp 3:** Routine + Catch-up 9m - < 15years **Comp 4:** Routine + Catch-up 9m - < 25years **Comp 5:** Routine + Catch-up>9m	**Dynamic transmission model with a treatment model.** V1, V2 + S, SR + IM, IQ + C + R + Treatment model **Treatment model:** Symptomatic cases + Inpatient + Outpatient + Death + Recovery **Time horizon:** 10 years	Outpatient, inpatient, vaccine supplies, routine and campaign vaccination costs	Operational cost of vaccination, probability of death, and hospitalization rate.	**Cases Averted (% reduction)**																		
											**Nairobi**				**Lwak**			**Delhi**			**Dong Thap**		**Kolkata**
					**Comp 1**						3642 (41)				254 (26)			8114 (38)			1275(24)		527 (18)
					**Comp 2**						5010 (57)				353 (36)			10723 (50)			979 (37)		926 (32)
					**Comp 3**						5971 (68)				526 (54)			12822 (60)			3201 (59)		1528 (53)
					**Comp 4**						6646 (76)				589 (60)			13872 (65)			3506 (65)		1818 (63)
					**Comp 5**						7124 (81)				680 (70)			16182 (76)			4049 (75)		2128 (74)
					**Deaths Averted (% reduction)**																		
											**Nairobi**				**Lwak**			**Delhi**			**Dong Thap**		**Kolkata**
					**Comp 1**						1 (33)				1 (25)			12 (39)			7 (25)		0 (0)
					**Comp 2**						1 (33)				1 (25)			16 (52)			10 (36)		0 (0)
					**Comp 3**						2 (67)				2 (50)			18 (58)			16 (57)		1 (100)
					**Comp 4**						2 (67)				2 (50)			20 (65)			18 (64)		1 (100)
					**Comp 5**						2 (67)				3 (75)			23 (74)			21 (75)		1 (100)
					**Comparison of five interventions ($/DALYs averted)**																		
											**Nairobi**			**Lwak**					**Delhi**		**Dong Thap**		**Kolkata**
					**Comp 1**						WD			WD					D		D		D
					**Comp 2**						3,141			WD					D		D		WD
					**Comp 3**						8,143			8,082					D		D		1,675
					**Comp 4**						11,925			13,213					D		CS		8,275
					**Comp 5**						31,344			22,561					CS		2,195		27,118
					**WD:** Weakly dominated, **CS:** Cost-Saving, **D:** Dominated																		
India,2021 [60]	**Base:** Do nothing **Comparator arm:** **St 1** Immunized at 6 months with 5-year vaccine efficacy. **St 2** Immunized at 6 months with 10-year vaccine efficacy. **St 3** Immunized at 6 months with 15-year vaccine efficacy	**Decision analytical model** (New-born cohort) **Decision tree model** with branches for: vaccine/no vaccine Treatment/ no treatment Uncomplicated/Severe/ Complicated Public/Private hospital **Time horizon:** 15 years	Outpatient, inpatient, out of pocket costs, vaccine supplies, and vaccination costs	Vaccine efficacy duration, typhoid incidence, inclusion vs exclusion of indirect costs, vaccine price.	**Urban** **Case reduction & deaths averted:** **Scenario 1** 5y = 17% & 17% **Scenario 2** 10y = 31% & 31% **Scenario 3** 15y = 36% & 36% **ICER: $/QALY gained w/o indirect costs:** **St 1:** 2,303 **St 2:** 939 **St 3:** 688 **ICER: $/QALY gained with indirect costs:** **St 1:** -1,055 **St 2:** -3,049 **St 3:** -3,683 **Rural** **Case reduction & deaths av.:** **Scenario 1** 5y = 19% **Scenario 2** 10y = 30% **Scenario 3** 15y = 36% **ICER: $/QALY gained w/o indirect costs:** **St 1**: 57,7771 **St 2**: 42,416 **St 3**: 40,026 **ICER: $/QALY gained with indirect costs:** **St 1**: 54,393 **St 2**: 38,413 **St 3:** 35,613																		
Low-income country, 2018 [39]	**Base:** Do nothing **Comp 1:** Routine vaccination at <1 year **Comp 2:** Routine + Catch-up 5–14 years **Comp 3:** Routine + Catch-up 1–14 years **Comp 4:** Routine + Catch-up 1–30 years	**Dynamic, age-structured, and** **deterministic compartmental model** S + I + R + C + V + Water (W) **Long cycle:** W to infect S **Short Cycle:** I + C infect S **Time horizon:** 10 years	Outpatient, inpatient, vaccine supplies, routine, school-based and community vaccination costs	Case-fatality rate, cost of vaccination, vaccine efficacy, duration of protection from vaccine, willingness-to-pay threshold, and carrier contribution	**Approximate incidence reduction**																		
					**Low endemicity (10 cases per 100,000**																		
					Comp 1 = 52%																		
					Comp 2 = 60%																		
					**Moderate endemicity (50 cases per 100,000)**																		
					Comp 1 = 65%																		
					Comp 2 = 75%																		
					**High endemicity (200 cases per 100,000)**																		
					Comp 1 = 55%																		
					Comp 2 = 50%																		
					**Cost-effectiveness**																		
					**Low endemicity (10 cases per 100,000)**																		
					**Strategy**				**ICER ($/DALY)**														
					Comp 1				11,631														
					Comp 2				15,854														
					Comp 3				21,023														
					Comp 4				53,773														
					**Moderate endemicity (50 cases per 100,000)**																		
					Comp 1			800															
					Comp 2			2,401															
					Comp 3			3,906															
					Comp 4			16,612															
					**High endemicity (200 cases per 100,000)**																		
					Comp 1					Cost-saving													
					Comp 2					Dominated													
					Comp 3					356													
					Comp 4					3,608													
Malawi, 2023 [61]	**Base:** Do nothing **Comp 1:** Routine vaccination at 9 months **Comp 2:** Routine + Catch-up 9m to <15years Coverage 85% increasing to 95% for routine, and for catch-up it was varied between 60–90%	**Age-specific stochastic model** S1, S2 + IA, IS + R + C + V1, V2. **Time horizon:** 10 years	Outpatient, inpatient, non-health seeking, vaccine supplies, and vaccination costs	Probability of death for inpatients, probability of hospitalization, incidence, and operational cost of vaccination	**Scenario 1: Outbreak occurs over the 10-year time horizon** **Comp 2:** $262/DALY, 67 DALYs averted per 100,000 people **Scenario 2: When no outbreak occurs** **Comp 2:** $770/DALY, 24 DALYs averted per 100,000 people **Scenario 3: When an outbreak has already occurred** **Comp 2:** $128/DALY, 282 DALYs averted per 100,000 people **NB:** Comp 1 dominated in all scenarios and number of cases for all vaccination related scenarios reduced number of cases and deaths																		
India, 2021 [62]	**Base:** Do nothing **Comp 1:** Routine vaccination at 9–12 months **Comp 2:** Routine + Community catch-up 1y to <15years **Comp 3:** Routine + School catch-up 5y to <15years and temp. Vaccination for 1-5y	**Dynamic transmission** **compartmental model** S + Ic + Is + R + C + V **Stratified by:** Age and geographical location **Time horizon:** 10 years	Healthcare costs (Adults & Children), OOP, Indirect costs, vaccine supplies, routine, school-based, and campaign vaccination costs.	Vaccination cost, case-fatality rate, and probability to become a carrier	**Incidence reduction** **Comp 1 Urban =** 66% **Comp 1 Urban + Rural =** 84% **Comp 2 Urban + Rural** = 95% **ICER ($/DALY)** **Comp 1 =** Dominated **Comp 2 in Urban + Rural =** 996 (Societal), 1942 (Payer) **Comp 3** = Dominated **NB:** Including campaign and school will reduce incidence and deaths in all scenarios. All strategies will be cost-saving by year 4 (Societal)																		
Lao PDR, 2023 [63]\\	**Base:** Do nothing **Comp 1:** school-based (SchB) 6-year-old **Comp 2:** Community-based (ComB), starting at 9 months of age **Comp 3:** ComB+catch-up at 15 years **Comp 4:** ComB+catch-up at 20 years **Comp 5:** SchB+catch-up at 20years	**Age-structured static decision tree model** with typhoid Infections. **Decision tree model with branches for:** Decision node with all the vaccine strategies No infection/ Infection/ Death Uncomplicated/ Complicated Healthy recovered/ Death **Time horizon:** Lifetime	Outpatient, Inpatient (with & without complications), non-medical, Indirect costs, vaccine supplies, school-based and campaign vaccination costs	Typhoid incidence rate	**Comp**	**Case Avoided**							**Deaths Avoided**										
					**1**	51.82							0.4										
					**Comp 2**	13.90							0.1										
					**Comp 3**	92.04							0.7										
					**Comp 4**	71.73							0.6										
					**Comp 5**	106.10							0.83										
						**ICER ($/QALY)**																	
					**Comp 1**	42,343																	
					**Comp 2**	80,133																	
					**Comp 3**	28,626																	
					**Comp 4**	40,881																	
					**Comp 5**	34,331																	
Bangladesh, 2024 [64]	**Base:** Do nothing **St1:** Campaign + Routine Dhaka and non-Dhaka **St2:** Campaign + Routine Dhaka **St3:** Campaign Dhaka+ Routine both **St4:** Routine All **St5:** Routine Dhaka **St6:** School-based & Routine both **St7:** School-based & Routine Dhaka **St8:** School-based Dhaka & Routine both **Routine:** 9-12m **Campaign:** Catch-up 1–15 years **School-based:** School children 5–15 years	**Dynamic transmission model** S + IC (Clinically infected), IS (Sub-clinically infected) + R + C + V, VS (Vaccinated with Sub-clinical infection) **Stratified:** Typhoid status (susceptible, clinically infected, sub-clinically infected, recovered, carrier, and vaccinated with sub-clinical infection), Dhaka/non-Dhaka, and age group (0-9m, 9- < 14m, 14- < 24m, 2- < 5y, 12 age groups from 5- < 65years of 5 years each, and >65years). **Time horizon:** 10 years	Healthcare costs (Adults & Pediatrics), indirect costs, vaccine supplies, school-based and campaign vaccination costs	Cost of illness adults, cost of illness paediatric, and typhoid incidence	**Cases averted (% Reduction)** **St1** = 3.77 million (79%) **St6** = 3.66 million (77%) **Discounted DALYs** **St1** = 275,000 **St2** = 50,000 **St3** = 187,000 **St4** = 166,000 **St5** = 28,000 **St6** = 267,000 **St7** = 49,000 **St8** = 186,000 **ICER (Societal)** **St1** = Lowest cost **St2-St8** = Dominated **ICER (Healthcare)** **St1** = $432/DALY averted **St2-St8** = Dominated																		
Afghanistan, Bangladesh, Benin, Burkina Faso, Burundi, Cambodia, Cameroon, Central African Republic, Chad, Comoros, DR Congo, Côte d’Ivoire, Djibouti, Eritrea, Ethiopia, Gambia, Ghana, Guinea, Guinea-Bissau, Haiti, India, Kenya, North Korea, Kyrgyzstan, Lao PDR, Lesotho, Liberia, Madagascar, Malawi, Mali, Mauritania, Mozambique, Myanmar, Nepal, Nicaragua, Niger, Nigeria, Pakistan, Papua New Guinea, Rwanda, São Tomé and Príncipe, Senegal, Sierra Leone, Solomon Islands, Somalia, South Sudan, Sudan, Tajikistan, Tanzania, Togo, Uganda, Yemen, Zambia and Zimbabwe, 2019 [25]	**Base:** Do nothing **Comp 1:** Routine vaccination at 9 months **Comp 2:** Routine + Catch-up <5years **Comp 3:** Routine + Catch-up 5 - < 15years	**Age-structured transmission dynamic** **model** S, SR + IM, IQ + R + C + V1, V2 **Time horizon:** 10 years	Outpatient, inpatient, vaccine supplies, routine and campaign vaccination costs	Probability of hospitalization, incidence of typhoid, and case fatality rate	**Projected cases averted** **Comp 1:** 33m cases (16–59) (30% reduction) **Comp 2:** 47m cases (22–73) (43% reduction) **Comp 3:** 63m cases (31–94) (57% reduction) **Projected death averted** **Comp 1:** 149,808 (26% reduction) **Comp 2:** 219,379 (38% reduction) **Comp 3:** 306,407 (53% reduction) **DALYs Averted (country with lowest to country with highest)** **Comp 1:** 0.046 to 1701 **Comp 2:** 0.069 to 2413 **Comp 3:** 0.098 to 3326 **Net costs in 000s (country with lowest to country with highest)** **Comp 1:** $-465 to $661,149 **Comp 2:** $-2,071 to $763,218 **Comp 3:** $-2,330–1,073,698 **Comp 3 was cost-effective in 49 of 52 countries if WTP = GDP** **NB:** Countries with high incidence of typhoid had the highest prob of C/E. High case-fatality rate and high treatment cost were a factor.																		
India, 2023 [65]	**Base:** Do nothing **Comp 1:** Routine vaccination at 9 months **Comp 2:** Routine + Catch-up 9m to <15years	**Model A:** Dynamic/Age-Structured/Deterministic States: S + I + R + C + V + W Environmental state: Yes **Model B:** Dynamic/Age-Structured/Deterministic States: S1 + I1 + R + S2 + I2 + C + V1 + V2 + W Environmental state: Yes **Model C:** Static, decision tree model States: N/A Environmental state: No **Model D:** Dynamic/Age-Structured/Deterministic States: S + I + R + C + V + W Environmental state: Yes **Model E:** Dynamic/Individual based/Stochastic States: U + S + Inc + AI/SI + C + W + V Environmental state: Yes **Time horizon:** 10 years	Healthcare costs (Adults & Pediatrics), out of pocket, indirect costs, vaccine supplies, routine, school-based, and campaign vaccination costs	Infectiousness of chronic carriers	**Median Cases averted** (unoptimistic and optimistic)																		
					**Comp 1: 10% - 46% and 2% - 16%**																		
					**Comp 2: 36% - 90% and 6% - 35%\**																		
					**ICER versus next best non-dominated alternative ($/DALY averted)**							**Sc 1**				**Sc2**	**Sc 3**			**Sc 4**		**Sc 5**	
					**Model A**		**1**					149				243	174			285		501	
							**2**					162				440	181			516		894	
					**Model B**		**1**					WD				WD	WD			WD		WD	
							**2**					147				209	167			242		431	
					**Model C**		**1**					WD				WD	WD			WD		WD	
							**2**					107				156	121			121		361	
					**Sc 1: 95% VE; 19yrs; 1% CFR** **Sc 2: 95% VE; 6yrs; 1% CFR** **Sc 3: 82% VE; 19yrs; 1% CFR** **Sc 4: 82% VE; 6yrs; 1% CFR** **Sc 5: 82% VE; 6yrs; 0.5% CFR** **NB: CFR = Case fatality rate, VE = Vaccine efficacy, WD = Weakly dominated**																		

## Discussion

This scoping review systematically mapped existing literature on the economic and public health impact of TCV, highlighting key findings, identifying research gaps, and summarizing the evidence. Of the 26 studies included in this review, a majority (n = 21) were from South Asia and Sub-Saharan Africa [25,36,44–46,48,50–56,58–62,57, 64-65]. This regional focus could be attributed to the significant public health burden of typhoid in these areas and the ongoing vaccination-related initiatives [2,66]. Notably, Latin America and parts of Asia, where typhoid is still endemic, were underrepresented in this review. This shows the need for further research in these regions to provide contextual evidence for vaccine decision-making.

Studies focusing on the COI of typhoid [44–53] highlighted its substantial economic burden, with reported costs varying across studies. Higher costs were reported for inpatient care when compared to outpatient care. Of the five studies that reported costs for both children and adults [44–46,50,51], only one reported the cost to be higher in children, which was attributed to longer hospitalization and, higher medical and indirect costs [51]. Studies adopting a societal perspective reported direct non-medical and indirect costs to account for 34–98% of total treatment costs [44–47,51–53], with two studies finding indirect costs to exceed direct medical costs [45,51]. This suggests that only employing the healthcare provider perspective may underestimate the true economic burden of the disease. Variation in cost estimates was further influenced by the addition of costs related to surgical procedures [44,46,49], the use of cost data from private or higher-tier hospitals [44,46,48,49] where the cost of treatment tends to be higher, and the regional differences in the prices of goods and services. Notably, only one study [46] included patients that had a drug resistant strain, reporting a median total outpatient cost of treating an extensively drug-resistant (XDR) case to be nearly 60% higher when compared to a non-XDR case, with the increased costs attributed to the use of more expensive antibiotics and more frequent healthcare facility visits. Conversely, the median total inpatient cost per case of treating an XDR case was about 20% lower than that for a non-XDR inpatient case. This difference was attributed to a higher prevalence of surgical interventions required to treat complications in the non-XDR cohort [46]. The reviewed studies demonstrated how the reported economic burden of typhoid fever varied based on methodological differences and local context, with the study perspective, hospital setting, disease severity, type of healthcare facility, geographical setting, and the presence of drug-resistant strains influencing the cost of treatment. Future studies should adopt both societal and healthcare payer perspectives, include drug-resistant cases where possible, ensure that patients from both urban and rural settings are included, and use local cost data to obtain a more accurate estimate of the economic burden. This will provide policymakers with context-specific evidence for decision making.

Studies reporting the cost of vaccination [54–57] and vaccine delivery [58] highlighted different factors influencing implementation costs across different settings. Variations in the cost of delivering TCV were associated with the vaccine coverage, vaccination strategy used (routine or campaign-based), logistical challenges, vaccine wastage rates, human resource costs, and regional differences in vaccine pricing. For instance, India reported a higher cost of vaccination than Malawi due to lower vaccine coverage in wealthy neighborhoods (vaccine hesitancy), higher price for the vaccine and supplies, and the need for additional personnel for mobilization [57]. The only study that compared the total cost of vaccination through both types of vaccination strategies, reported marginally lower costs for vaccination through routine immunization when compared to campaigns [55]. Vaccine delivery costs varied based on specific activities included, such as community outreach, cold chain maintenance, adverse event monitoring, and healthcare worker training, as well as broader systemic factors including population characteristics, vaccine hesitancy, and healthcare capacity. The primary drivers for start-up costs during the introduction period included micro-planning, capacity building, and social mobilization [54,57,58]. Introducing TCV through a campaign that is integrated with other interventions can help minimize incremental costs [58]. As countries transition out of Gavi support [67], future studies on the cost of vaccination and vaccine delivery should report economic costs rather than only financial costs to help capture the full value of resources consumed. This will provide policymakers with the evidence needed to guide decisions on sustainable vaccine financing.

Dynamic models were used in the two public health impact studies [43,59] and five economic evaluations [25,36,39,62,64,65] to simulate changes in typhoid transmission, population structure, and immunity levels over time. Dynamic models are preferred over static models because they effectively capture the time-dependent changes associated with typhoid transmission and the indirect effects of vaccination [68]. However, the models [43,59] relied on assumptions for parameters like typhoid transmission rates, antimicrobial resistance, vaccine coverage, vaccine hesitancy, and vaccine efficacy. To address these gaps, countries could strengthen surveillance systems, while further research can be conducted to ascertain the level of vaccine hesitancy, and the efficacy of the vaccine in the local population.

The nine economic evaluations [25,36,39,60–65] identified in this scoping review reflect the growing interest in assessing the value for money of TCV, especially given its recent introduction and adoption. Despite the novelty of TCV, the emerging work on economic evaluations suggests strong interest from researchers and policymakers, as highlighted by the number of CEAs included in this review compared to the two identified in the previous scoping review by Luthra et al [24].

Variations in the reported ICERs across the economic evaluations were primarily influenced by the vaccine strategy employed and the incidence of typhoid. TCV was shown to be more cost-effective in high-incidence and urban settings, where the disease burden is greater, population density is higher, and sanitation infrastructure is often inadequate. Chauhan et al. highlighted this difference, reporting TCV to be cost-saving in urban areas but not cost-effective in rural settings [60]. Four studies further emphasized this benefit of TCV by reporting it to be cost-saving in urban areas, where the reduction in treatment costs outweighed the costs associated with vaccination [36,39,60,62]. These findings suggest that densely populated urban settings may offer the greatest potential for cost-effective vaccine strategies.

In terms of vaccine delivery strategy, the review found that combining routine vaccination alongside catch-up campaigns targeting broader age groups was generally reported to be more cost-effective than routine vaccination alone [25,36,61,62,64]. However, some studies identified routine vaccination as the most cost-effective strategy in specific scenarios [36,39,65]. These findings highlight the importance of evaluating both routine and campaign-based vaccination strategies, as the most cost-effective approach depends on contextual factors such as the disease burden, population density, and costs. A subset of studies that reported ICERs from both societal and healthcare provider perspectives showed that the ICERs were significantly lower when the societal perspective was used, primarily due to the inclusion of indirect costs, which were substantial [60,62,64]. The findings suggest that adopting a societal perspective in future CEAs can provide a more comprehensive understanding with regards to the economic benefits of vaccination with TCV.

Sensitivity analyses identified several key parameters that the ICER was sensitive to, including typhoid incidence rates, vaccine efficacy, coverage levels, case-fatality rates, and treatment costs. Higher incidence rates improved cost-effectiveness by increasing the number of cases averted [60], while greater vaccine efficacy and higher coverage enhanced the health gains from vaccination [69]. High case-fatality rates and high treatment costs also improved the cost-effectiveness of vaccination by highlighting the vaccine’s potential to prevent deaths and reduce health-related expenditures.

A notable finding of this review was the consistent underrepresentation of key parameters. Herd immunity was largely not incorporated, likely because most studies were published prior to real-world data on the indirect benefits of TCV being available in published literature. This omission could have led to an underestimation of the broader public health and economic benefits of TCV [70]. Similarly, the impact of TCV on antimicrobial resistance (AMR) was insufficiently explored in the cost-effectiveness studies, with only the two modeling studies explicitly considering this parameter [43,59] and only one COI reporting the cost of treating patients infected by a drug resistant strain [46]. Given the rising prevalence of multidrug-resistant *S. Typhi* and the associated treatment challenges [71], incorporating AMR-related parameters into future models would be essential for accurately capturing the full economic value of TCV.

Several areas for future research have been highlighted in this review. First, the use of a lifetime horizon should be explored as it will highlight the long-term economic and public health benefits of TCV, which is essential to inform sustainable immunization policies. Second, there is a need for models to integrate parameters such as herd immunity and AMR, and account for sub-regional differences in typhoid incidence within countries. Third, more primary-level cost data for vaccine delivery and program implementation in diverse settings are required to refine cost-effectiveness analyses. Lastly, evaluating the equity implications through an extended cost-effectiveness analysis [72], may provide a better understanding of the broader social and economic impact of TCV and this can support more equitable and informed health policy decisions.

This scoping review had its limitations. Although we did not apply language restrictions in our database search, we only included articles published in English, so studies reported in other languages may have been missed. We did not use formal quality appraisal tools for the cost of illness, cost of vaccination, or public health impact studies, and therefore could not evaluate the methodological rigor of individual analyses. We also did not search the grey literature, which may have led to the omission of unpublished program evaluations or reports.

## Conclusion

This review summarized the evidence available in literature on the economic and public health impact of TCV, and It highlights the growing evidence generated from regions with a high typhoid burden. Majority of the studies showed TCV to be cost-effective, especially in urban settings that have a high incidence of typhoid. The review highlighted gaps, especially around the effect of AMR, herd immunity, and long-term impact. Future research should be conducted in diverse settings, use the societal perspective, and include model parameters that can help describe the impact of drug-resistant strains and herd immunity. This will help provide a clearer and more complete understanding of TCV’s value. Strengthening of surveillance systems and local research will help provide contextual evidence to policymakers that can be used for decision-making around sustainable immunization programs.

## Supporting information

S1 Appendix Detailed search strategy. Search strategy used across PubMed, Web of Science, HTA, Scopus, and NHS EED databases to identify studies.
(DOCX)

S2 Appendix PRISMA-ScR checklist. Completed PRISMA-ScR checklist showing compliance with scoping review reporting guidelines, with corresponding manuscript page numbers. Source: The PRISMA-ScR checklist is reproduced from Tricco AC, et al. PRISMA Extension for Scoping Reviews (PRISMA-ScR): Checklist and Explanation. Ann Intern Med. 2018;169(7):467–473. Licensed under Creative Commons Attribution 4.0 International License (CC BY 4.0).
(DOCX)

S3 Appendix Data extraction template. Data extraction form to capture study ID, vaccine strategy, model type, model parameters, cost of illness, cost of vaccination, and key findings.
(DOCX)

S4 Appendix Drummond checklist. Checklist used to assess the methodological quality of economic evaluations, rated on a scale of 1–10.
(DOCX)

S5 Appendix Quality assessment results. Results of the quality assessment of cost-effectiveness analyses included in the study, that were assessed using the Drummond checklist and Doran scoring system.
(DOCX)

S1 Table Comprehensive data extraction table. Table that consolidates all extracted study data, including unadjusted cost values, before outcome grouping.
(XLSX)
