# Supplementary material for: Examining epidemiological models and economic analyses of typhoid conjugate vaccine: A scoping review
Source: PLOS Glob Public Health. 2026 Mar 30;6(3):e0005162. doi: 10.1371/journal.pgph.0005162 (PMC13035140; doi:10.1371/journal.pgph.0005162)
Supplement: S1 Appendix — Search strategy used across PubMed, Web of Science, HTA, Scopus, and NHS EED databases to identify studies. (DOCX) [file pgph.0005162.s001.docx]

**S1 Appendix. Detailed search strategy**

| **#1** | *PubMed:*  ((((((((((("Cost*") OR ("Cost-effectiveness")) OR ("Economic evaluation")) OR ("Cost-benefit")) OR (“Cost effective*")) OR (“Cost-effective*")) OR (“Cost-utility”)) OR ("Cost utility")) OR ("Cost analys*")) OR (“Cost-analys*”)) OR ("Cost benefit")) |
| --- | --- |
|  | *Web of Science:* ("Cost*" OR "Cost-effectiveness" OR "Economic evaluation" OR "Cost-benefit" OR “Cost effective*" OR “Cost-effective*" OR “Cost-utility” OR "Cost utility" OR "Cost analys*" OR “Cost-analys*” OR "Cost benefit") |
|  | *HTA:* ("Cost*" OR "Cost-effectiveness" OR "Economic evaluation" OR "Cost-benefit" OR “Cost effective*" OR “Cost-effective*" OR “Cost-utility” OR "Cost utility" OR "Cost analys*" OR “Cost-analys*” OR "Cost benefit") |
|  | *Scopus:* (TITLE-ABS-KEY("Cost*") OR TITLE-ABS-KEY("Cost-effectiveness") OR TITLE-ABS-KEY("Economic evaluation") OR TITLE-ABS-KEY("Cost-benefit") OR TITLE-ABS-KEY (“Cost effective*") OR TITLE-ABS-KEY (“Cost-effective*") OR TITLE-ABS-KEY (“Cost-utility”) OR TITLE-ABS-KEY ("Cost utility") OR TITLE-ABS-KEY ("Cost analys*") OR TITLE-ABS-KEY (“Cost-analys*”) OR TITLE-ABS-KEY ("Cost benefit")) |
|  | *NHS EED:* (Cost OR Cost-effectiveness OR Economic evaluation OR Cost-benefit OR Cost effective OR Cost-effective OR Cost-utility OR Cost utility OR Cost analyses OR Cost-analysis OR Cost benefit) |
| **#2** | *PubMed:* (((((("Model*") OR ("Dynamic model")) OR ("Mathematical model")) OR ("Population Model")) OR ("Epidemiological model")) OR ("Stochastic model")) OR ("Static model") OR ("Transmission model") |
|  | *Web of Science:* ("Model*" OR "Dynamic model" OR "Mathematical model" OR "Population Model" OR "Epidemiological model" OR "Stochastic model" OR "Static model" OR "Transmission model") |
|  | *HTA: ("Model*" OR "Dynamic model" OR "Mathematical model" OR "Population model" OR "Epidemiological model" OR "Stochastic model" OR "Static model" OR "Transmission model")* |
|  | *Scopus:* (TITLE-ABS-KEY("Model*") OR TITLE-ABS-KEY("Dynamic model") OR TITLE-ABS-KEY("Mathematical model") OR TITLE-ABS-KEY("Population model") OR TITLE-ABS-KEY("Epidemiological model") OR TITLE-ABS-KEY("Stochastic model") OR TITLE-ABS-KEY("Static model") OR TITLE-ABS-KEY("Transmission model")) |
|  | *NHS EED:* (Model* OR Dynamic model OR Mathematical model OR Population model OR Epidemiological model OR Stochastic model OR Static model OR Transmission model) |
| **#3** | *PubMed:* ("Typhoid vaccin*") OR ("Typhoid conjugate vaccine") OR ("TCV") |
|  | *Web of Science: ("Typhoid vaccin*" OR "Typhoid conjugate vaccine" OR "TCV")* |
|  | *HTA:* ("Typhoid vaccin*" OR "Typhoid conjugate vaccine" OR "TCV") |
|  | *Scopus:* (TITLE-ABS-KEY("Typhoid vaccin*") OR TITLE-ABS-KEY("Typhoid conjugate vaccine") OR TITLE-ABS-KEY("TCV")) |
|  | *NHS EED:* (Typhoid vaccin* OR Typhoid conjugate vaccine OR TCV) |
| **Final query** | #1 OR #2 AND #3 |
