# Supplementary material for: Examining epidemiological models and economic analyses of typhoid conjugate vaccine: A scoping review
Source: PLOS Glob Public Health. 2026 Mar 30;6(3):e0005162. doi: 10.1371/journal.pgph.0005162 (PMC13035140; doi:10.1371/journal.pgph.0005162)
Supplement: S3 Appendix — Data extraction form to capture study ID, vaccine strategy, model type, model parameters, cost of illness, cost of vaccination, and key findings. (DOCX) [file pgph.0005162.s003.docx]

**S3 Appendix. Microsoft Excel template for data extraction**

| **STUDY NAME** | **VACCINE STRATEGY** | **EPIDEMIOLOGICAL MODEL** | **HERD IMMUNITY** | **MODEL PARAMETER** | **CURRENCY, YEAR** | **COST OF ILLNESS** | **COST OF VACCINATION** | **STUDY FINDINGS** |
| --- | --- | --- | --- | --- | --- | --- | --- | --- |
