# Supplementary material for: Examining epidemiological models and economic analyses of typhoid conjugate vaccine: A scoping review
Source: PLOS Glob Public Health. 2026 Mar 30;6(3):e0005162. doi: 10.1371/journal.pgph.0005162 (PMC13035140; doi:10.1371/journal.pgph.0005162)
Supplement: S4 Appendix — Checklist used to assess the methodological quality of economic evaluations, rated on a scale of 1–10. (DOCX) [file pgph.0005162.s004.docx]

**S4 Appendix. Drummond checklist for assessing the quality of cost-effectiveness analyses**

| **Questions** |
| --- |
|  |
| 1. Was a well-defined question posed in answerable form? |
| 2. Was a comprehensive description of the competing alternatives given? |
| 3. Was the effectiveness of the programme established? |
| 4. Were all the important and relevant costs and consequences for each alternative identified? |
| 5. Were costs and consequences measured accurately in appropriate physical units? |
| 6. Were costs and consequences valued credibly? |
| 7. Were costs and consequences adjusted for differential timing? |
| 8. Was an incremental analysis of costs and consequences of alternatives performed? |
| 9. Was allowance made for uncertainty in the estimates of costs and consequences? |
| 10. Did the presentation and discussion of study results include all issues of concern to users? |
| **Quality level** |
|  |
| **KEY:** The checklist can be used to rate the quality of an economic evaluation on a scale of 1–10, with  1–3 points indicating poor quality, 4–7 points indicating average quality, and 8–10 points indicating good quality. |
