# Supplementary material for: Examining epidemiological models and economic analyses of typhoid conjugate vaccine: A scoping review
Source: PLOS Glob Public Health. 2026 Mar 30;6(3):e0005162. doi: 10.1371/journal.pgph.0005162 (PMC13035140; doi:10.1371/journal.pgph.0005162)
Supplement: S5 Appendix — Results of the quality assessment of cost-effectiveness analyses included in the study, that were assessed using the Drummond checklist and Doran scoring system. (DOCX) [file pgph.0005162.s005.docx]

|  | Antillon et al | Bilcke et al | Burrows et al | Chauhan et al | Lo NC et al | Phillips et al | Ryckman et al | Soukavong et al | Weyant et al |
| --- | --- | --- | --- | --- | --- | --- | --- | --- | --- |
| 1. Was a well-defined question posed in answerable form? | 1 | 1 | 1 | 1 | 1 | 1 | 1 | 1 | 1 |
| 1. Was a comprehensive description of the competing alternatives given? | 1 | 1 | 1 | 1 | 1 | 1 | 1 | 1 | 1 |
| 1. Was the effectiveness of the programme established? | 1 | 1 | 1 | 1 | 1 | 1 | 1 | 1 | 1 |
| 1. Were all the important and relevant costs and consequences for each alternative identified? | 1 | 1 | 1 | 1 | 1 | 1 | 1 | 1 | 1 |
| 1. Were costs and consequences measured accurately in appropriate physical units? | 1 | 1 | 1 | 1 | 1 | 1 | 1 | 1 | 1 |
| 1. Were costs and consequences valued credibly? | 1 | 1 | 1 | 1 | 1 | 1 | 1 | 1 | 1 |
| 1. Were costs and consequences adjusted for differential timing? | 1 | 1 | 1 | 1 | 1 | 1 | 1 | 1 | 1 |
| 1. Was an incremental analysis of costs and consequences of alternatives performed? | 1 | 1 | 1 | 1 | 1 | 1 | 1 | 1 | 1 |
| 1. Was allowance made for uncertainty in the estimates of costs and consequences? | 1 | 1 | 1 | 1 | 1 | 1 | 1 | 0 | 1 |
| 1. Did the presentation and discussion of study results include all issues of concern to users? | 1 | 1 | 1 | 1 | 1 | 1 | 1 | 1 | 1 |
| **Total** | 10 | 10 | 10 | 10 | 10 | 10 | 10 | 9 | 10 |
| **Quality level** | GOOD | GOOD | GOOD | GOOD | GOOD | GOOD | GOOD | GOOD | GOOD |
| **KEY:** The checklist can be used to rate the quality of an economic evaluation on a scale of 1–10, with 1–3 points indicating poor quality, 4–7 points indicating average quality, and 8–10 points indicating good quality. | | | | | | | | | |

**S5 Appendix. Quality assessment of included cost-effectiveness analyses using the Drummond checklist and Doran scoring system.**
